# Supplementary material for: In vivo partial reprogramming of myofibers promotes muscle regeneration by remodeling the stem cell niche
Source: Nat Commun. 2021 May 25;12:3094. doi: 10.1038/s41467-021-23353-z (PMC8149870; doi:10.1038/s41467-021-23353-z)
Supplement: Supplementary file 1 — Supplementary Information [file 41467_2021_23353_MOESM1_ESM.pdf]

In vivo partial reprogramming of myofibers promotes muscle regeneration by remodeling the stem cell niche

Chao Wang[1], Ruben Rabadan Ros[1], Paloma Martinez Redondo[1], Zaijun Ma[1], Lei Shi[1], Yuan Xue[1], Isabel Guillen-Guillen[1], Ling Huang[2], Tomoaki Hishida[1], Hsin-Kai Liao[1], Estrella Nuñez Delicado[3], Concepcion Rodriguez Esteban[1], Pedro Guillen Garcia[4], Pradeep Reddy[1]and Juan Carlos Izpisua Belmonte[1,*]

[1] Gene Expression Laboratory, Salk Institute for Biological Studies, La Jolla, CA 92037, USA
[2] Integrative Genomics and Bioinformatics Core, Salk Institute for Biological Studies, La Jolla, CA 92037, USA
[3] Universidad Católica San Antonio de Murcia (UCAM), Campus de los Jerónimos, Nº 135 12 Guadalupe 30107, Spain
[4] Department of Traumatology and Research Unit, Clinica CEMTRO, 28035 Madrid, Spain

*Correspondence should be addressed to J.C.I.B. (belmonte@salk.edu)

- Supplementary Figure 1-11
- Supplementary Table 1

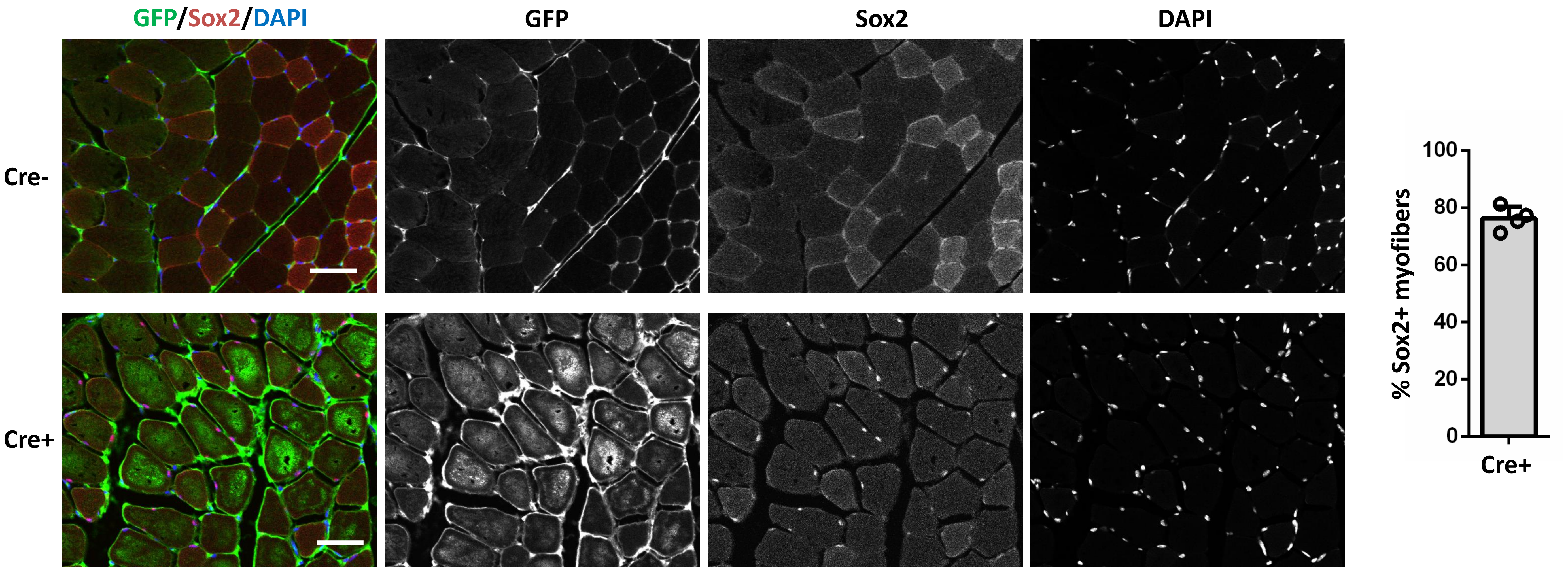

Supplementary Fig. 1: Immunostaining of GFP and Sox2 and quantification of GFP⁺ myofibers in EDL muscles with myofiber-specific OSKM induction.
Scale bars= 50 µm. Error bars represent mean+SD of 4 mice.

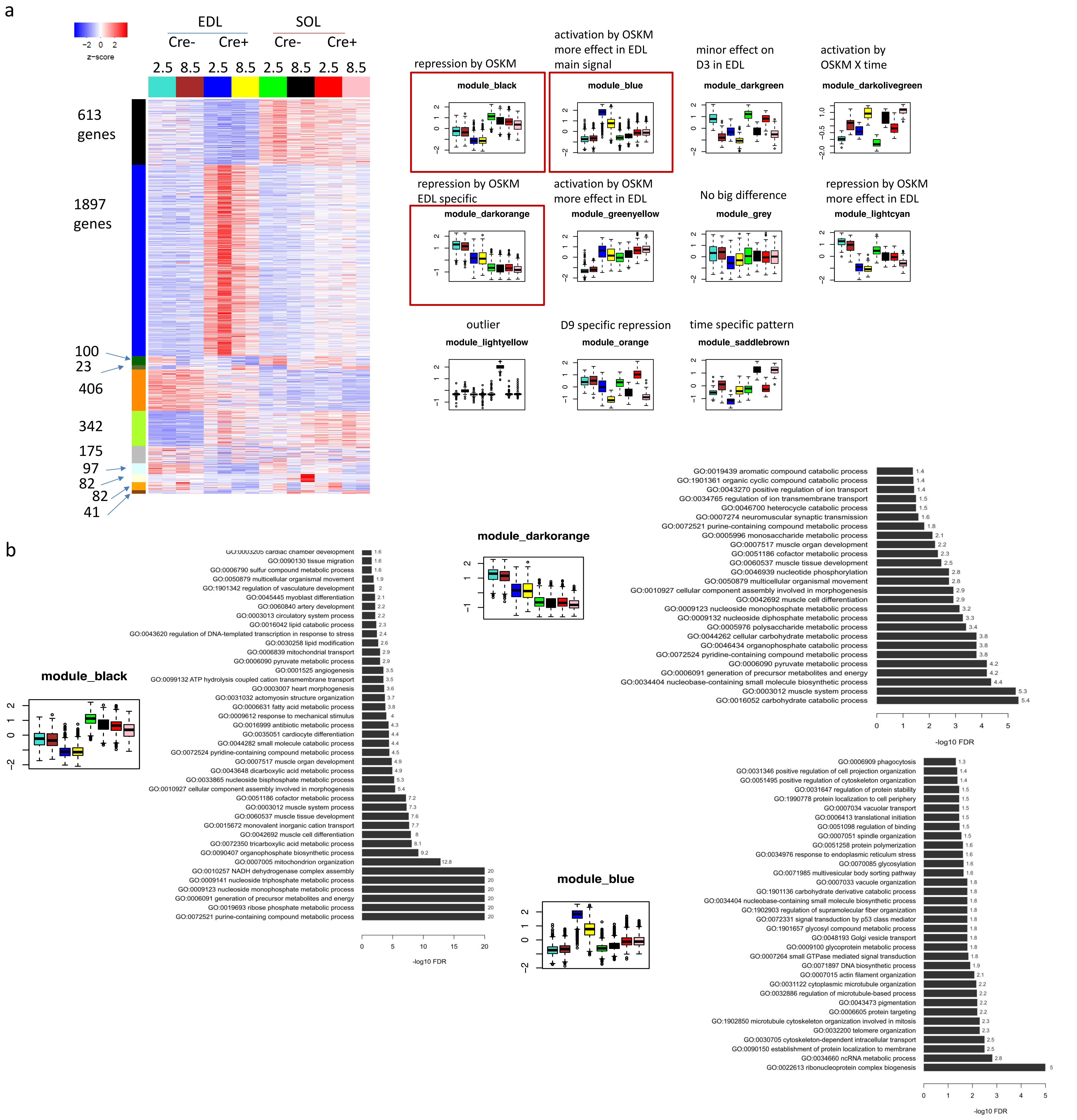

Supplementary Fig. 2: Pattern analysis of DE genes from both EDL and SOL muscles with myofiber-specific OSKM induction.
a, Heatmap of DE genes from both EDL and SOL muscles. n=2 independent biological samples. 11 modules identified from pattern analysis. The lower bounds, center, and upper bounds of box indicated 25th, 50th, and 75th percentile of the values. The whiskers extended the box to 1.5 fold of Interquartile Range or the minimum and maximum values. Dots outside of the whiskers indicated potential outliers. b, Enriched GO terms in biological process of the 3 modules outlined in graph a. n=2 independent biological samples.

a

D2.5

Gene Set	Description	NES	P Value	FDR
mmu00190	Oxidative phosphorylation	-2.5727	0	0
mmu05012	Parkinson disease	-2.4353	0	0
mmu04714	Thermogenesis	-2.4071	0	0
mmu04932	Non-alcoholic fatty liver disease (NAFLD)	-2.3631	0	0
mmu05016	Huntington disease	-2.2639	0	0
mmu04723	Retrograde endocannabinoid signaling	-2.0633	0	0.00025859
mmu00640	Propanoate metabolism	-1.8616	0.0074074	0.0084226
mmu05010	Alzheimer disease	-1.8241	0	0.011055
mmu00020	Citrate cycle (TCA cycle)	-1.7945	0.0037313	0.013619

D8.5

Gene Set	Description	NES	P Value	FDR
mmu04932	Non-alcoholic fatty liver disease (NAFLD)	-2.2104	0	0
mmu05016	Huntington disease	-2.2206	0	0
mmu04714	Thermogenesis	-2.3268	0	0
mmu05012	Parkinson disease	-2.3458	0	0
mmu00190	Oxidative phosphorylation	-2.5179	0	0
mmu00020	Citrate cycle (TCA cycle)	-1.9533	0	0.0034297
mmu00640	Propanoate metabolism	-1.9403	0	0.003451
mmu05010	Alzheimer disease	-1.9142	0	0.0055918
mmu01200	Carbon metabolism	-1.8336	0	0.01511

b

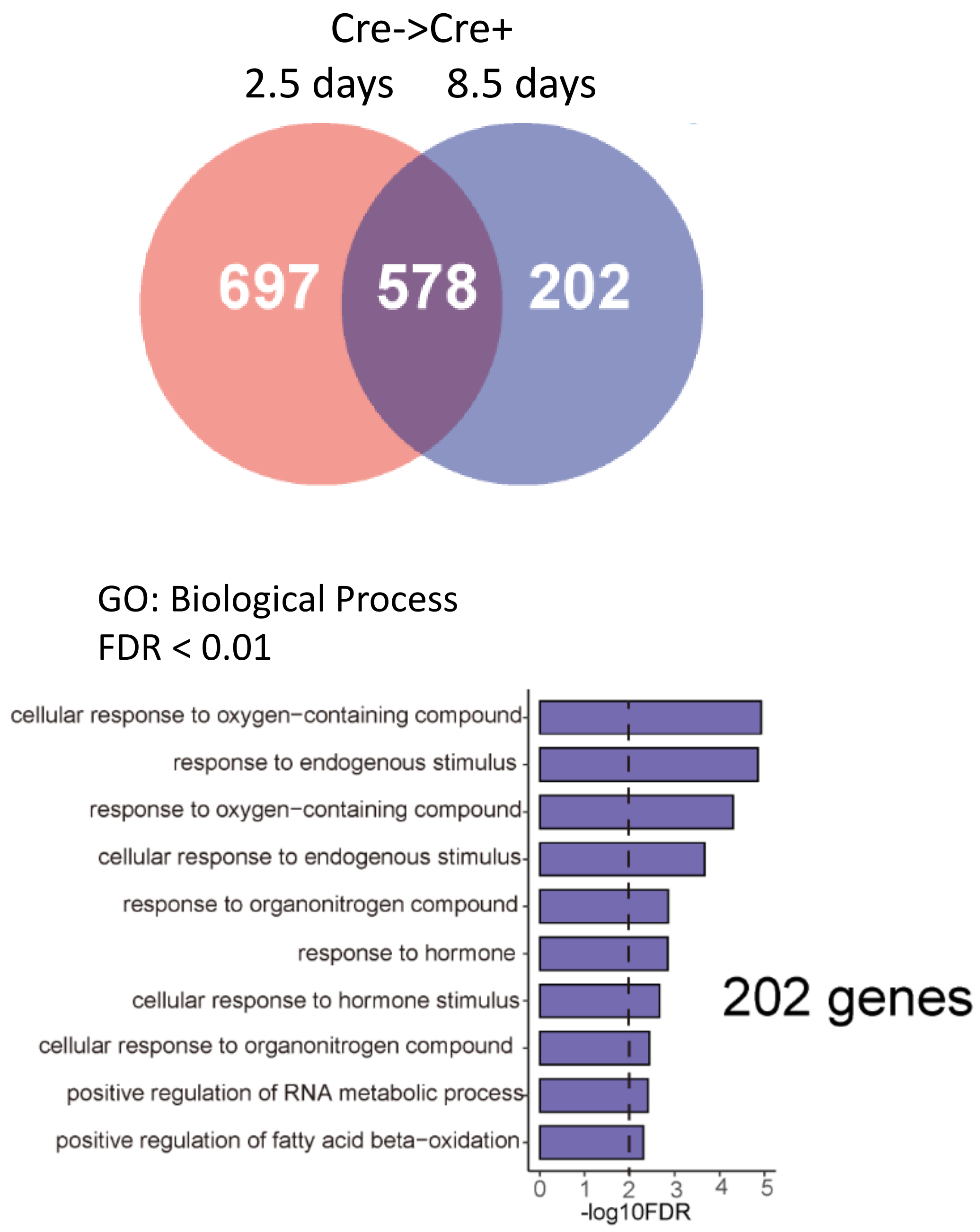

Supplementary Fig. 3: Annotations of DE genes from EDL muscles with myofiber-specific OSKM induction.
a, Gene set enrichment analysis (GSEA) of the transcriptome of EDL muscles after 2.5- or 8.5-days Dox treatment. b, Enriched GO terms in biological process of genes specifically downregulated in EDL muscles after 8.5-days Dox treatment.

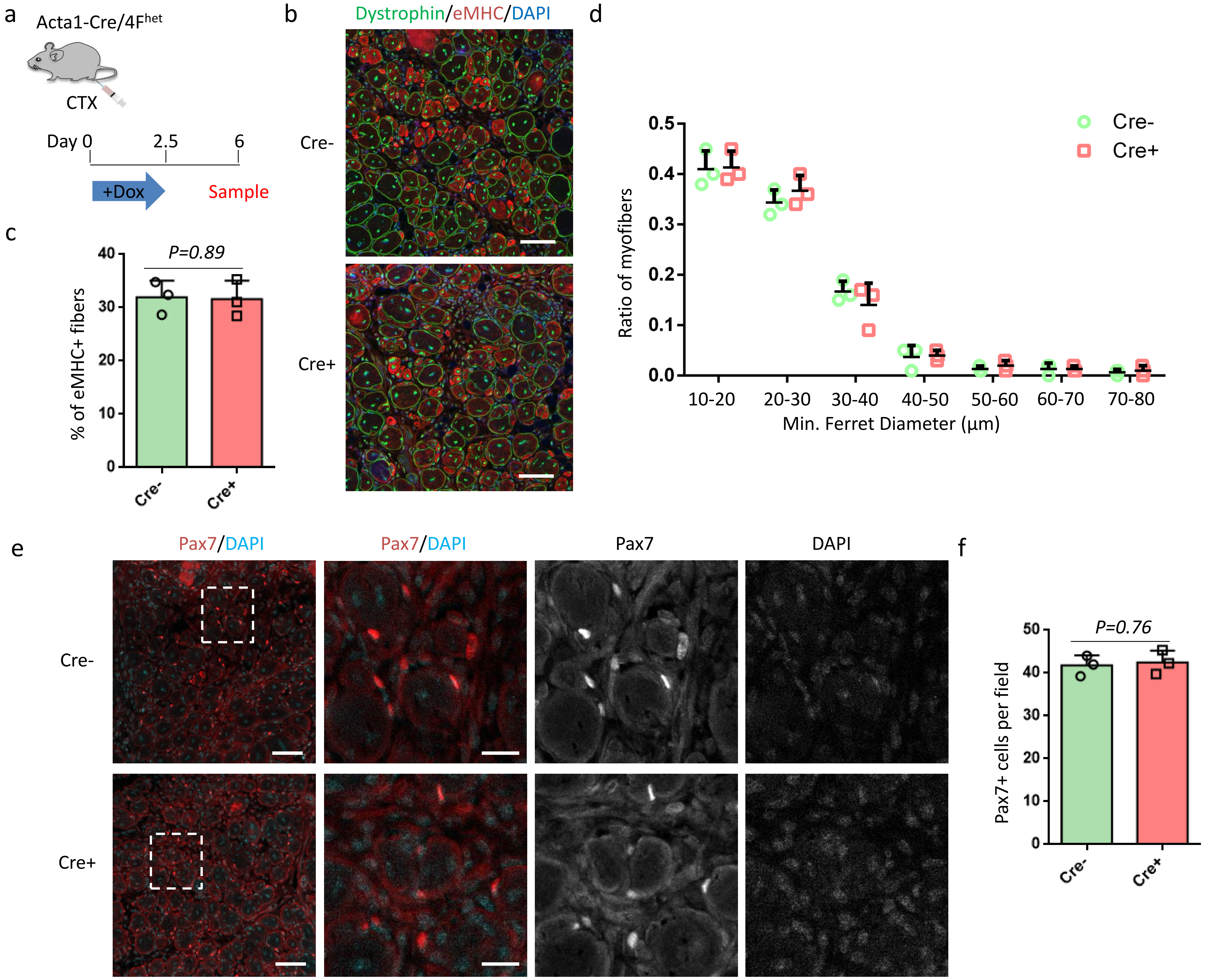

Supplementary Fig. 4: OSKM induction post-injury does not accelerate muscle regeneration.
a, Schematic representation of the experimental design. b, Immunostaining of embryonic myosin heavy chain (eMHC) and Dystrophin in TA muscle sections. Scale bars= 50 µm. c, Quantification of the percentage of immature myofibers that express eMHC. d, Myofiber size distributions in TA muscle sections. e, Immunostaining of Pax7 in TA muscle sections. Scale bars= 50 µm. Representative regions are shown at higher magnification. Scale bars= 25 µm. f, Quantification of Pax7^{+} cells in TA muscle sections. Error bars represent mean+SD of 3 mice. A two-sided unpaired Student's t-test was performed.

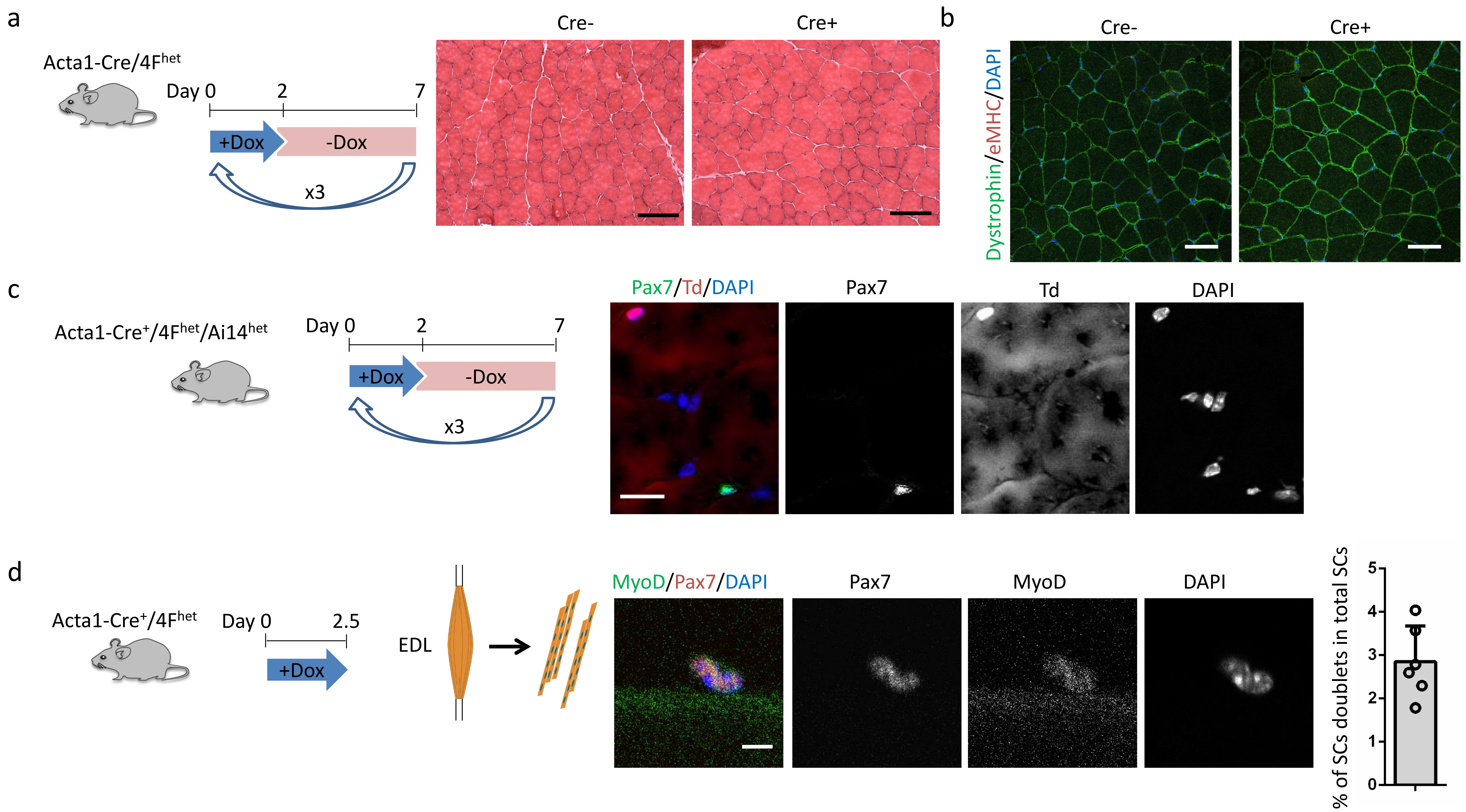

Supplementary Fig. 5: Cyclic OSKM induction in myofiber does not change the myofiber morphology but induces SCs doublets.
a, H&E staining of TA muscle sections after cyclic Dox treatment. Scale bars= 100 µm. Similar results were repeated independently in 3 pairs of mice. In addition, similar results were repeated twice for each mouse. b, Immunostaining of embryonic myosin heavy chain (eMHC) and Dystrophin in TA muscle sections. Scale bars= 50 µm. Similar results were repeated independently in 3 pairs of mice. In addition, similar results were repeated twice for each mouse. c, Immunostaining of Pax7 in TA muscle sections of Acta1-Cre^{+}/4F^{het}/Ai14^{het} mice. Scale bars= 25 µm. Similar results were repeated independently in 3 pairs of mice. In addition, similar results were repeated twice for each mouse. d, SCs doublets identified in Cre^{+} myofibers with Immunostaining of Pax7 and MyoD. Error bars represent mean+SD of 5 mice. Scale bars= 10 µm.

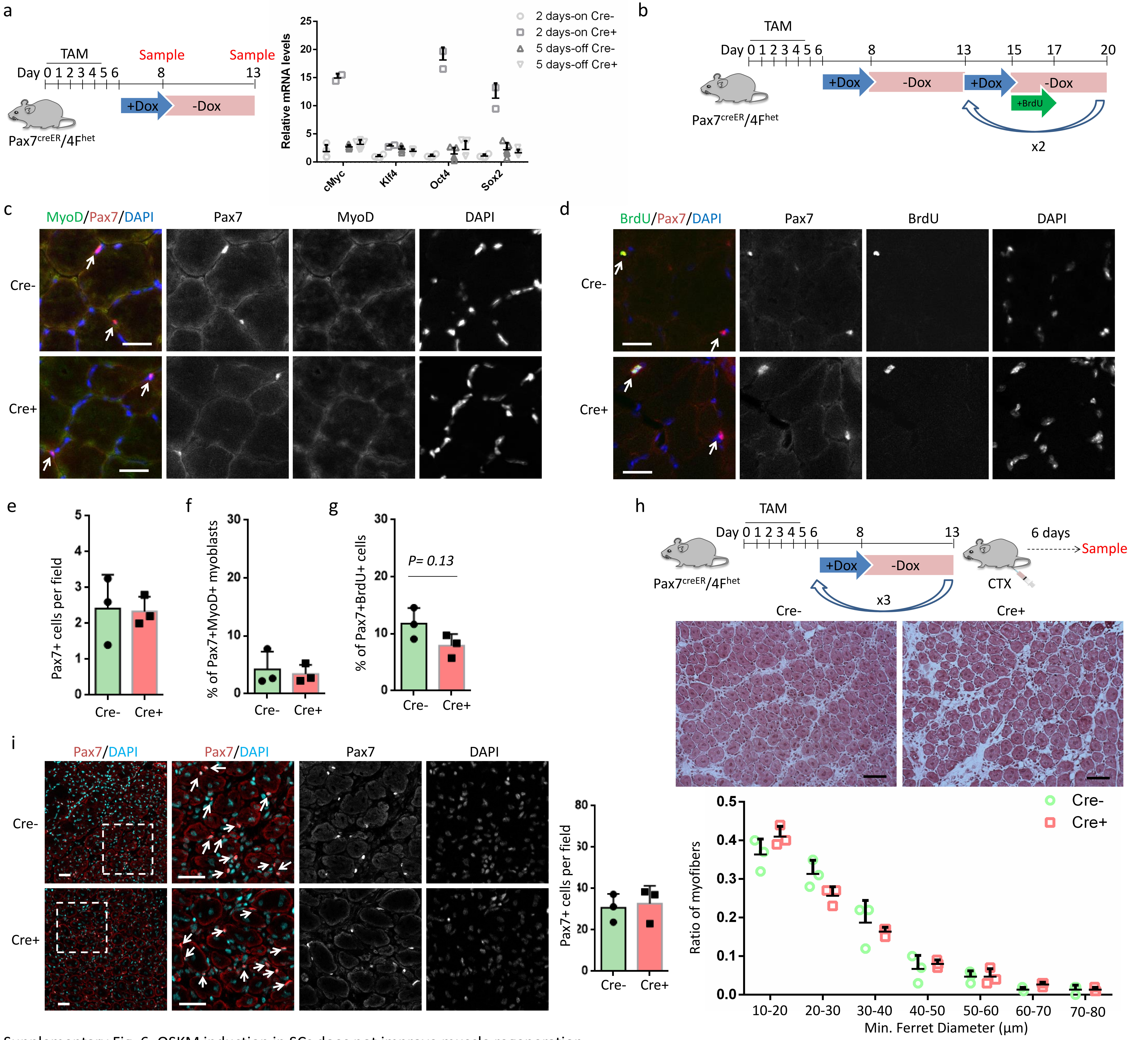

Supplementary Fig. 6: OSKM induction in SCs does not improve muscle regeneration.

a, Relative RNA levels of OSKM in Pax7$^{creER/+}$/4F^{het} (as Cre+ for simplify) and Pax7$^{+/+}$/4F^{het} (as Cre-) mice. b, Schematic representation of the experimental design. c, Immunostaining of Pax7 and BrdU in TA muscle sections. Scale bars= 25 µm. d, Immunostaining of Pax7 and MyoD in TA muscle sections. Pax7^{+} cells are indicated by arrows. Scale bars= 25 µm. e, Quantification of Pax7^{+} cells per field. f, Quantification of the percentage of Pax7^{+} cells that express MyoD. g, Quantification of the percentage of Pax7^{+} cells with BrdU signals. h, H&E staining of TA muscle sections and myofiber size distributions in TA muscle sections. Scale bars= 100 µm. i, Immunostaining and quantification of Pax7 in TA muscle sections. Representative regions are shown at higher magnification. Pax7^{+} cells are indicated by arrows. Scale bars= 50 µm. Error bars represent mean+SD of 3 mice. A two-sided unpaired Student’s t-test was performed.

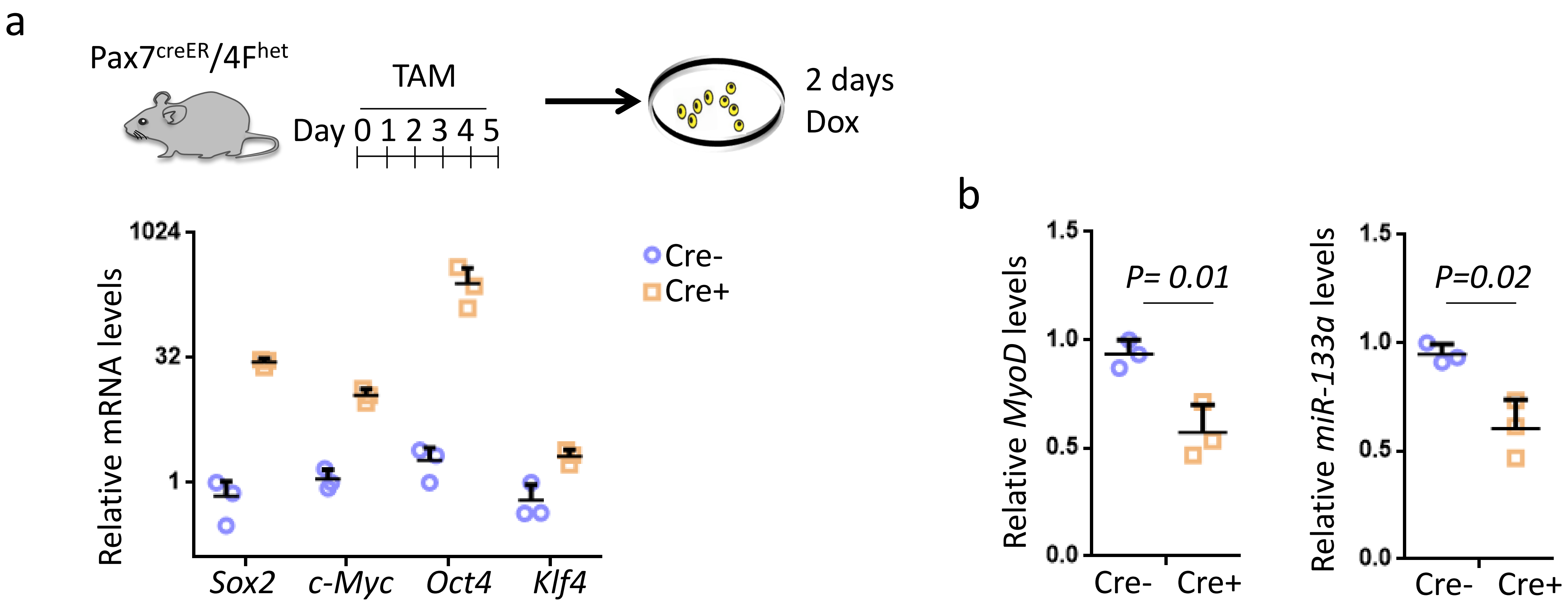

Supplementary Fig. 7: Gene expressions in primary myoblasts

a, Relative RNA levels of OSKM in primary myoblasts isolated from Pax7$^{creER/+}$/4F^{het} and Pax7$^{+/+}$/4F^{het} mice. b, Relative level of *MyoD* and *miR-133a* in primary myoblasts isolated from Pax7^{creER}/4F^{het} mice. Error bars represent mean+SD of 3 biological replicates. A two-sided unpaired Student's t-test was performed.

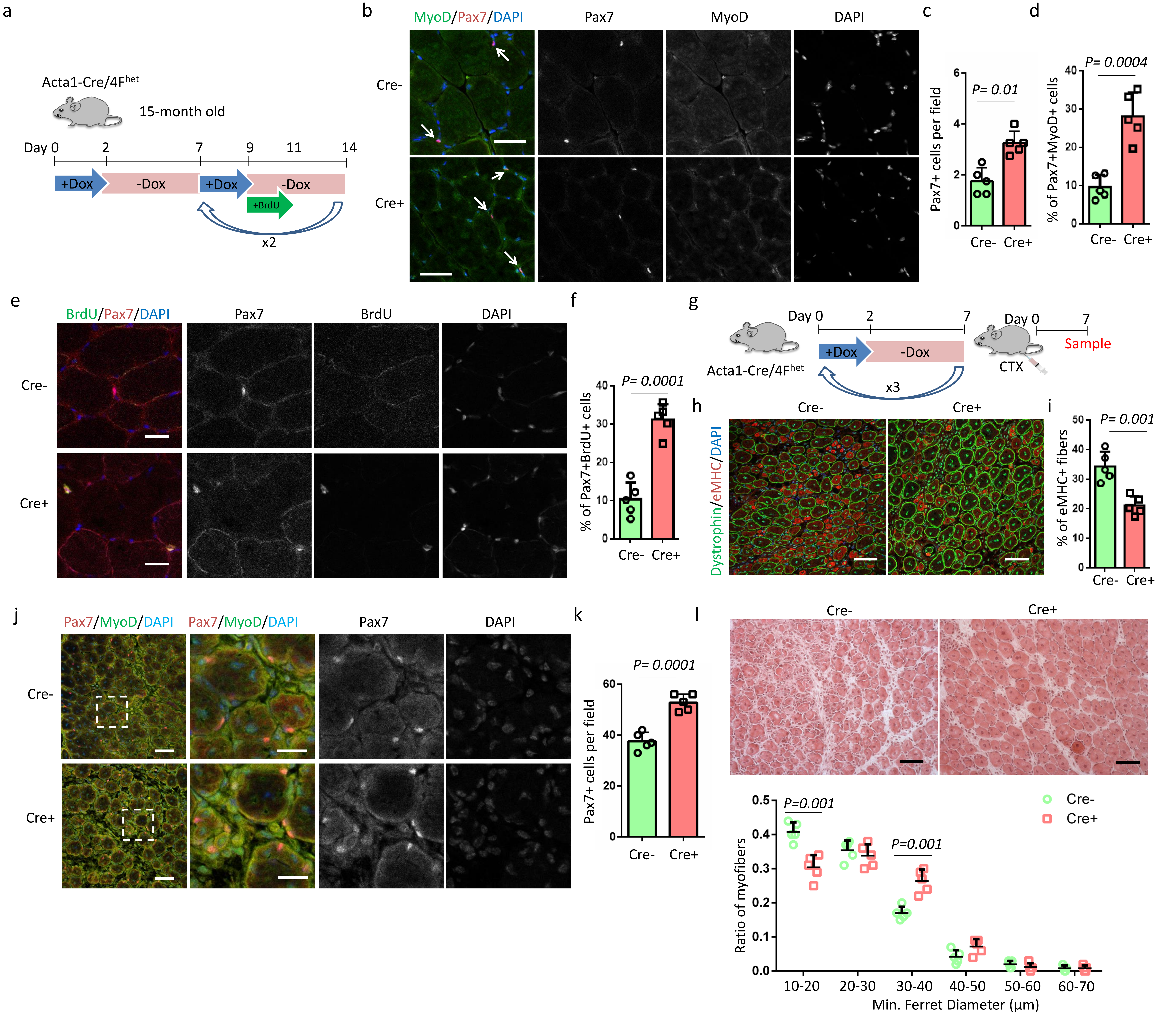

Supplementary Fig. 8: Myofiber-specific OSKM induction accelerates muscle regeneration in aging mice.

a, Schematic representation of the experimental design. b-d, Immunostaining of Pax7 and MyoD and quantification of Pax7^{+} cells per field and the percentage of Pax7^{+}MyoD^{+} cells in TA muscle sections. Pax7^{+} cells are indicated by arrows. Scale bars= 50 μm. e and f, Immunostaining of Pax7 and BrdU and the quantification of the percentage of Pax7^{+}BrdU^{+} cells in TA muscle sections. Scale bars= 10 μm. g, Schematic representation of the experimental design. h and i, Immunostaining of embryonic myosin heavy chain (eMHC) and Dystrophin in TA muscle sections, and the quantification of the percentage of immature myofibers that express eMHC. Scale bars= 50 μm. j and k, Immunostaining of Pax7 and MyoD, and quantification of Pax7^{+} cells in TA muscle sections. Scale bars= 50 μm. Representative regions are shown at higher magnification. Scale bars= 25 μm. Arrows indicate Pax7^{+} cells. l, H&E staining of TA muscle sections and myofiber size distributions in TA muscle sections. Scale bars= 100 μm. Error bars represent mean+SD of 5 mice. A two-sided unpaired Student's t-test was performed.

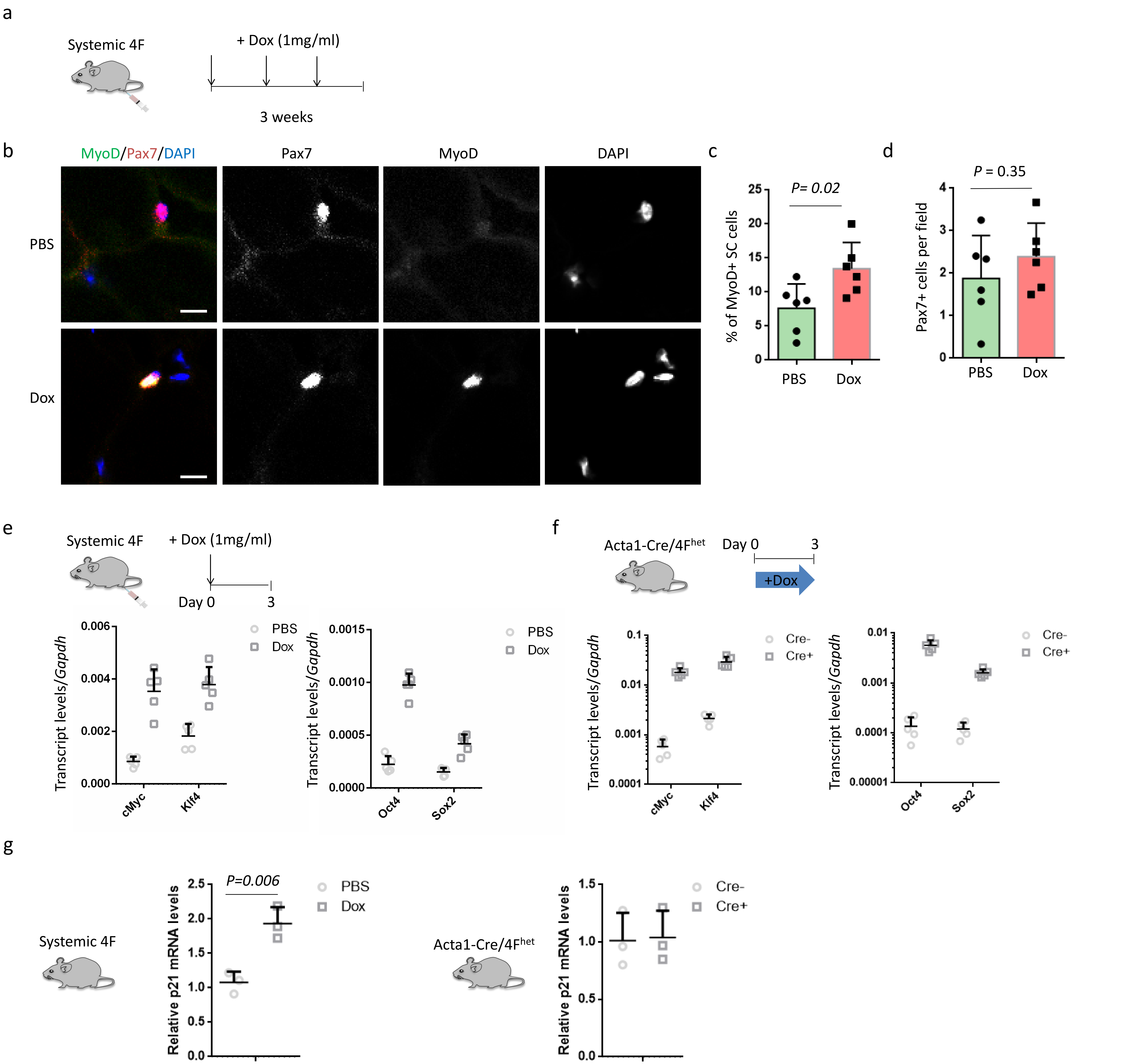

Supplementary Fig. 9: The number and state of SCs in TA muscles of Systemic 4F mice.

a, Schematic representation of the experimental design. b-d, Immunostaining of Pax7 and MyoD and quantification of Pax7$^+$ cells per field and the percentage of Pax7$^+$MyoD$^+$ cells in TA muscle sections. Scale bars= 10 µm. Error bars represent mean+SD of 6 mice. e, The levels of OSKM in TA muscles of systemic 4F mice after local Dox or PBS injection. Error bars represent mean+SD of 5 mice. f, The levels of OSKM in TA muscles of Acta1-Cre$^+$/4F^{het} mice. Error bars represent mean+SD of 5 mice. For e and f, we used the same Gapdh primer in our previous publication[8]. g, The levels of *p21* in SCs of systemic 4F mice and Acta1-Cre$^+$/4F^{het} mice. Error bars represent mean+SD of 3 mice. A two-sided unpaired Student's t-test was performed.

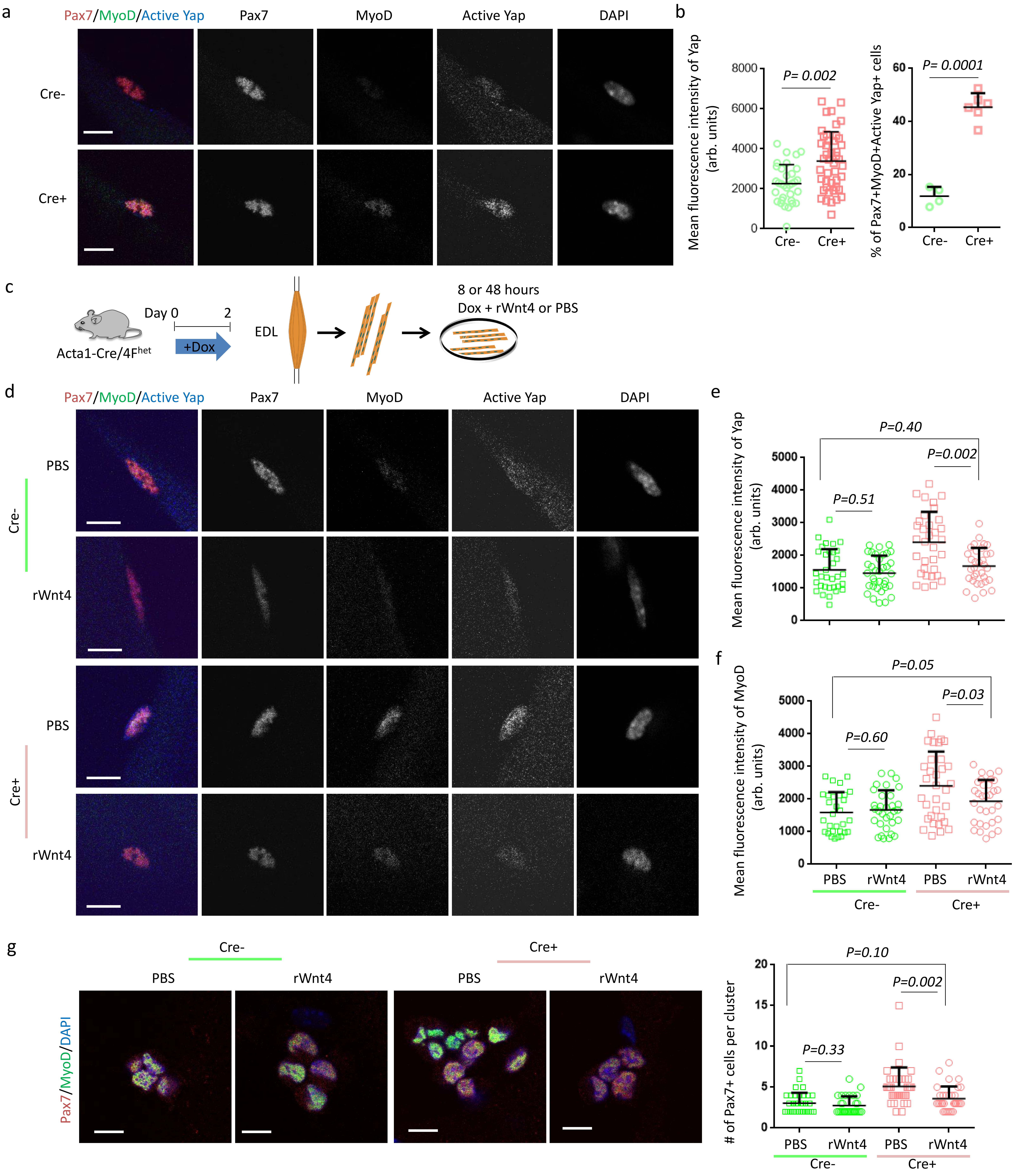
a
Pax7/MyoD/Active Yap
Pax7
MyoD
Active Yap
DAPI
Cre-
Cre+
b
Mean fluorescence intensity of Yap (arb. units)
P= 0.002
8000
6000
4000
2000
0
Cre-
Cre+
% of Pax7+MyoD+Active Yap+ cells
P= 0.0001
60
40
20
0
Cre-
Cre+
c
Day 0
2
+Dox
Acta1-Cre/4F^het
EDL
8 or 48 hours
Dox + rWnt4 or PBS
d
Pax7/MyoD/Active Yap
Pax7
MyoD
Active Yap
DAPI
PBS
rWnt4
Cre-
PBS
rWnt4
Cre+
e
Mean fluorescence intensity of Yap (arb. units)
P=0.40
P=0.002
P=0.51
5000
4000
3000
2000
1000
0
f
Mean fluorescence intensity of MyoD (arb. units)
P=0.05
P=0.03
P=0.60
5000
4000
3000
2000
1000
0
PBS
rWnt4
PBS
rWnt4
Cre-
Cre+
g
Cre-
Cre+
PBS
rWnt4
PBS
rWnt4
Pax7/MyoD/DAPI
# of Pax7+ cells per cluster
P=0.10
P=0.002
P=0.33
20
15
10
5
0
PBS
rWnt4
PBS
rWnt4
Cre-
Cre+

Supplementary Fig. 10: Recombinant Wnt4 diminishes the activation and proliferation of SCs on myofibers with OSKM induction.
a, Immunostaining of Pax7, MyoD and active Yap in single myofibers. Scale bars= 10 µm. b, Quantification of the signal intensity of active Yap. n= 33 SCs on Cre- myofibers and 45 SCs on Cre+ myofibers. Quantification of the percentage of $Pax7^{+}MyoD^{+}$active Yap^{+} cells. n=4 Cre- muscles and 6 Cre+ muscles. Error bars represent mean+SD. c, Schematic representation of the experimental design. d, Immunostaining of Pax7, MyoD and active Yap in single myofibers after culture for 8 hours with or without rWnt4 treatment. Scale bars= 10 µm. e, Quantification of the signal intensity of active Yap. n= 33 SCs and 35 SCs on Cre- myofibers treated with PBS and rWnt4, respectively. n= 33 SCs and 33 SCs on Cre+ myofibers treated with PBS and rWnt4, respectively. Error bars represent mean+SD. f, Quantification of the signal intensity of MyoD. n= 30 SCs and 32 SCs on Cre- myofibers treated with PBS and rWnt4, respectively. n= 33 SCs and 31 SCs on Cre+ myofibers treated with PBS and rWnt4, respectively. Error bars represent mean+SD. g, Immunostaining of Pax7 and MyoD and quantification of $Pax7^{+}$ cells per cluster in single myofibers after culture for 48 hours with or without rWnt4 treatment. n= 35 and 30 cell clusters on Cre- myofibers treated with PBS and rWnt4, respectively. n= 37 and 33 cell clusters on Cre+ myofibers treated with PBS and rWnt4, respectively. Myofibers were isolated from of 4 Cre^{-} EDL muscles and 4 Cre^{+} EDL muscles. Error bars represent mean+SD. A two-sided unpaired Student’s t-test was performed.

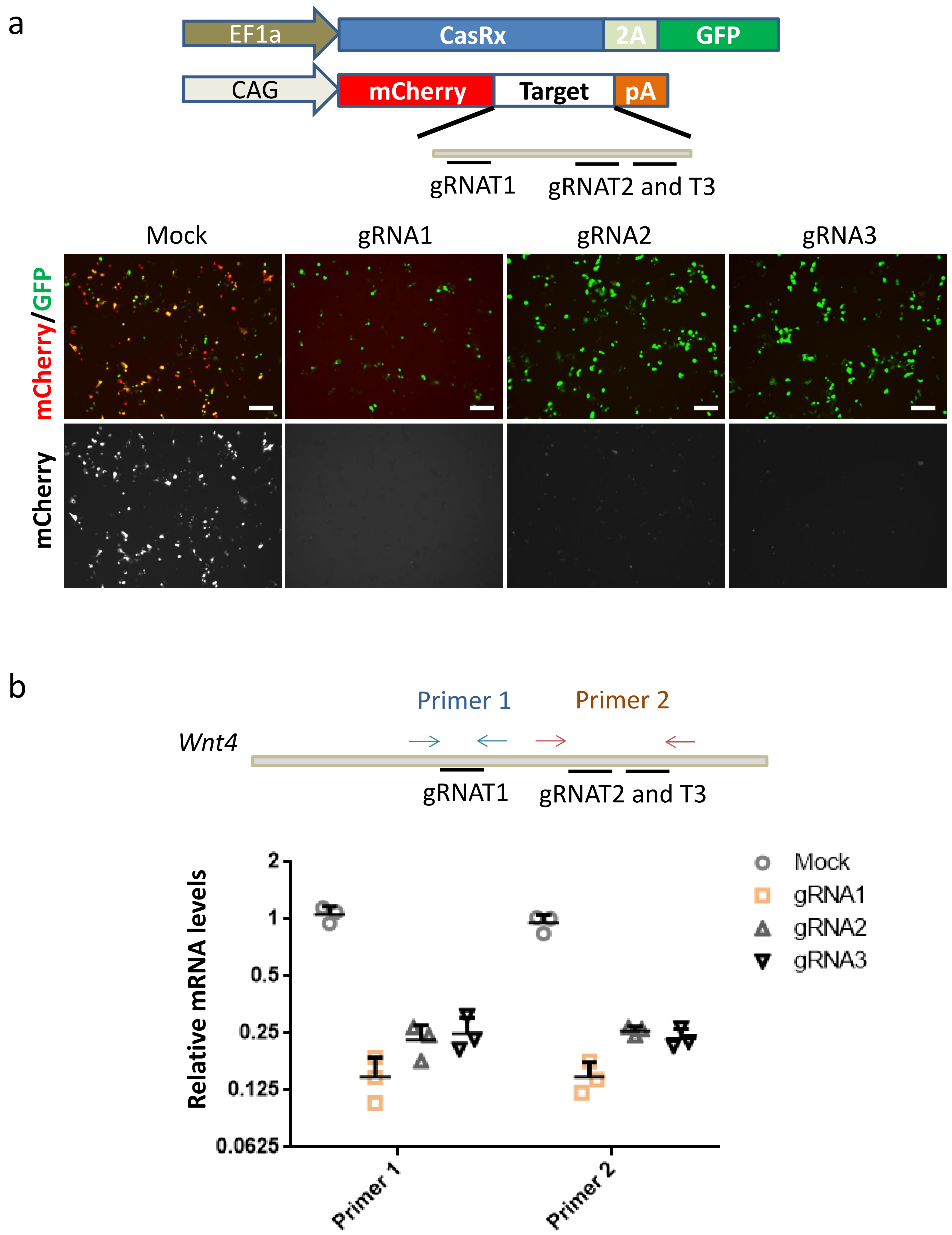

Supplementary Fig. 11: Screen gRNAs for CasRx-mediated Wnt4 knockdown.
a, Illustration of the florescence reporter designed for detecting the knockdown efficiency of CasRx-related gRNAs. Scale bars= 100 µm. b, Quantification of the knockdown efficiency of CasRx-related gRNAs. Error bars represent mean+SD of 3 biological replicates. A two-sided unpaired Student's t-test was performed.

Supplementary Table 1: qPCR primers

Gene Name	5'-Sequence-3'
Oct4-f	GGCTTCAGACTTCGCCTTCT
Oct4-r	TGGAAGCTTAGCCAGGTTCG
Sox2-f	TTTGTCCGAGACCGAGAAGC
Sox2-r	CTCCGGGAAGCGTGTACTTA
Klf4-f	GCACACCTGCGAACTCACAC
Klf4-r	CCGTCCCAGTCACAGTGGTAA
c-Myc-f	ACCACCAGCAGCGACTCTGA
c-Myc-r	TGCCTCTTCTCCACAGACACC
Gapdh-f	CATGGCCTTCCGTGTTCCTA
Gapdh-r	CCTGCTTCACCACCTTCTTGAT
Wnt4-f	CGAGGAGTGCCAATACCAGT
Wnt4-r	GCCACACCTGCTGAAGAGAT
p21-f	CGCTGTCTTGCACTCTGGT
p21-r	CGTTTTCGGCCCTGAGATGTT
Wnt4 Primer1-f	CTGGAGAAGTGTGGCTGTGA
Wnt4 Primer1-r	CAGCCTCGTTGTTGTGAAGA
Wnt4 Primer2-f	TGCGAGGTAAAGACGTGCTG
Wnt4 Primer2-r	CTTGAACTGTGCATTCCGAGG
TATA Proximal-f	CAATTCCCAGCACCAAAAGT
TATA Proximal-r	TCCAAATAAGGTAGGTACCCAAAG
