# Supplementary material for: In vivo partial reprogramming of myofibers promotes muscle regeneration by remodeling the stem cell niche
Source: Nat Commun. 2021 May 25;12:3094. doi: 10.1038/s41467-021-23353-z (PMC8149870; doi:10.1038/s41467-021-23353-z)
Supplement: Supplementary file 3 — Reporting Summary [file 41467_2021_23353_MOESM3_ESM.pdf]

nature research

Corresponding author(s): Juan Carlos Izpisua Belmonte

Last updated by author(s): Mar 25, 2021

Reporting Summary

Nature Research wishes to improve the reproducibility of the work that we publish. This form provides structure for consistency and transparency in reporting. For further information on Nature Research policies, see our Editorial Policies and the Editorial Policy Checklist.

Statistics

For all statistical analyses, confirm that the following items are present in the figure legend, table legend, main text, or Methods section.

n/a	Confirmed	
☐	☒	The exact sample size (*n*) for each experimental group/condition, given as a discrete number and unit of measurement
☐	☒	A statement on whether measurements were taken from distinct samples or whether the same sample was measured repeatedly
☐	☒	The statistical test(s) used AND whether they are one- or two-sided<br>*Only common tests should be described solely by name; describe more complex techniques in the Methods section.*
☐	☒	A description of all covariates tested
☐	☒	A description of any assumptions or corrections, such as tests of normality and adjustment for multiple comparisons
☐	☒	A full description of the statistical parameters including central tendency (e.g. means) or other basic estimates (e.g. regression coefficient) AND variation (e.g. standard deviation) or associated estimates of uncertainty (e.g. confidence intervals)
☐	☒	For null hypothesis testing, the test statistic (e.g. *F*, *t*, *r*) with confidence intervals, effect sizes, degrees of freedom and *P* value noted<br>*Give P values as exact values whenever suitable.*
☒	☐	For Bayesian analysis, information on the choice of priors and Markov chain Monte Carlo settings
☒	☐	For hierarchical and complex designs, identification of the appropriate level for tests and full reporting of outcomes
☒	☐	Estimates of effect sizes (e.g. Cohen's *d*, Pearson's *r*), indicating how they were calculated

Our web collection on statistics for biologists contains articles on many of the points above.

Software and code

Policy information about availability of computer code

Data collection	ZEISS Zen software (v2.3, blue edition) was used to perform confocal imaging.
Data analysis	We used ImageJ (version 1.52i) for digital image analysis, and Graphpad Prism (version 7) for data plotting and statistical analysis.<br>For RNA-seq analysis: Raw reads were aligned to the mm10 genome using STAR [v2.5.3a]; The number of reads uniquely aligned to RefSeq exons were quantified by HOMER [v4.9.1]; Differential expression (DE) analysis was performed on the raw reads using DESeq2 [v1.22.2].

For manuscripts utilizing custom algorithms or software that are central to the research but not yet described in published literature, software must be made available to editors and reviewers. We strongly encourage code deposition in a community repository (e.g. GitHub). See the Nature Research guidelines for submitting code & software for further information.

Data

Policy information about availability of data

All manuscripts must include a data availability statement. This statement should provide the following information, where applicable:

- Accession codes, unique identifiers, or web links for publicly available datasets
- A list of figures that have associated raw data
- A description of any restrictions on data availability

The original data of RNA-seq are deposited to GEO dataset (GSE148911, https://www.ncbi.nlm.nih.gov/geo/query/acc.cgi?acc=GSE148911). All data supporting the findings of this study are available from the corresponding authors upon reasonable request.

Field-specific reporting

Please select the one below that is the best fit for your research. If you are not sure, read the appropriate sections before making your selection.

- [x] Life sciences
- [ ] Behavioural & social sciences
- [ ] Ecological, evolutionary & environmental sciences

For a reference copy of the document with all sections, see nature.com/documents/nr-reporting-summary-flat.pdf

Life sciences study design

All studies must disclose on these points even when the disclosure is negative.

Sample size	The samples sizes for each experiment are indicated in the figure legends. No statistical method was used to predetermine sample size. Sample size was chosen based on the sample availability and statistic relevance. Sample size was at least n=3 independent biological replicates for in vivo and in vitro experiments, except for RNA-seq analysis which had two biological replicates.
Data exclusions	No data were excluded from this study.
Replication	In vivo experiments were replicated in at least 3 mice. In vitro experiments were replicated at least 3 times in independent studies. We were under same experimental conditions to replicate the study and all replicates were successful with similar results.
Randomization	Samples were allocated randomly.
Blinding	Investigators were blinded to mice genotypes during analysis of in vivo experiment. Investigators knew the genotype of the cells for in vitro studies, as primary cells were isolated from mice with known genotypes.

Reporting for specific materials, systems and methods

We require information from authors about some types of materials, experimental systems and methods used in many studies. Here, indicate whether each material, system or method listed is relevant to your study. If you are not sure if a list item applies to your research, read the appropriate section before selecting a response.

Materials & experimental systems

n/a	Involved in the study	
☐	☒	Antibodies
☐	☒	Eukaryotic cell lines
☒	☐	Palaeontology and archaeology
☐	☒	Animals and other organisms
☒	☐	Human research participants
☒	☐	Clinical data
☒	☐	Dual use research of concern

Methods

n/a	Involved in the study	
☒	☐	ChIP-seq
☒	☐	Flow cytometry
☒	☐	MRI-based neuroimaging

Antibodies

Antibodies used	Immunostaining: Anti-Pax7 (DSHB, Pax7-c), anti-MyoD (SCBT, sc-377460, G-1), anti-BrdU (Abcam, ab6326, [BU1/75 (ICR1)]), anti-Sox2 (Cell Signaling, #2748), anti-Oct-3/4 (SCBT, sc-5279, C-10), anti-GFP (SCBT, sc-9996, B-2) and anti-active Yap (Abcam, ab205270, EPR19812) were diluted at a ratio 1:200. Anti-eMHC (DSHB, F1.652) was diluted at a ratio 1:80. Dystrophin (Abcam, ab15277) antibody was diluted at a ratio 1:500. Goat anti-Mouse IgG1 (Alexa Fluor 568, A-21124), Goat anti-Mouse IgG2b (Alexa Fluor 488, A-21141), Goat anti-Mouse IgG2b (Alexa Fluor 647, A-21242), Donkey anti-Rabbit IgG (H+L) (Alexa Fluor 488, A-21206) and Donkey anti-Rat IgG (H+L) (Alexa Fluor 488, A-21208) were from Thermo Fisher Scientific. All the secondary antibodies were diluted at a ratio 1:500.<br>Western blots: Anti-p53 (Cell Signaling, #2524S) was diluted at a ratio of 1:100. Anti-p21 (Abcam, ab109520) and anti-Gapdh (Cell Signaling, #2188S) were diluted at a ratio of 1:1000. Anti-mouse IgG, HRP-linked (Cell Signaling, #7076) and anti-Rabbit IgG, HRP-linked (Cell Signaling, #7074) were diluted at a ratio of 1: 2500.<br>Chromatin Immunoprecipitation: 1 µg anti-FLAG antibody (Sigma, F1804, M2) was used per sample.
Validation	Various antibody dilutions were tested according to manufacturer's recommended dilution. All the antibodies used in this study have been used and reported in prior studies.<br>Anti-Pax7 (DSHB, Pax7-c) has been cited in at least 97 references such as an immunostaining application in mouse muscle tissues: Lineage Tracing Reveals a Subset of Reserve Muscle Stem Cells Capable of Clonal Expansion under Stress. Cell stem cell 24.6 (2019 Jun 6): 944-957.e5.<br>Anti-MyoD (SCBT, sc-377460, G-1) has been cited in 51 references such as an immunostaining application in mouse muscle tissues (PMID: # 32183151).<br>Anti-BrdU (Abcam, ab6326, [BU1/75 (ICR1)]) has been cited in 1228 references such as an immunostaining application in mouse muscle tissues: Lineage Tracing Reveals a Subset of Reserve Muscle Stem Cells Capable of Clonal Expansion under Stress. Cell stem

cell 24.6 (2019 Jun 6): 944-957.e5.
Anti-Sox2 (Cell Signaling, #2748) and anti-Oct-3/4 (SCBT, sc-5279, C-10) has been been verified in our previous publication: In Vivo Amelioration of Age-Associated Hallmarks by Partial Reprogramming. Cell 167, 1719–1733.
Anti-GFP (SCBT, sc-9996, B-2) has been cited in 2617 reference such as an immunostaining application in PMID: 33720931.
Anti-active Yap (Abcam, ab205270, EPR19812) has been cited in at least 20 reference such as an immunostaining application in PMID: 32449258.
Anti-eMHC (DSHB, F1.652) has been cited in at least 64 references such as an immunostaining application in skeletal muscle: Skeletal muscle 9.1 (2019 Aug 14): 22.
Dystrophin (Abcam, ab15277) antibody and secondary antibodies including Goat anti-Mouse IgG1 (Alexa Fluor 568, A-21124), Goat anti-Mouse IgG2b (Alexa Fluor 488, A-21141), Goat anti-Mouse IgG2b (Alexa Fluor 647, A-21242), Donkey anti-Rabbit IgG (H+L) (Alexa Fluor 488, A-21206) and Donkey anti-Rat IgG (H+L) (Alexa Fluor 488, A-21208) have been verified in PMID: 28752107.
Anti-p53 (Cell Signaling, #2524S) has been cited in at least 62 references such as an application in western blots: PMID: 30415165.
Anti-p21 (Abcam, ab109520) has been cited in at least 198 references such as an application in western blots: PMID: 32064156.
Anti-Gapdh (Cell Signaling, #2188S) has been cited in at least 3448 references such as an application in western blots: PMID: 33230114.
Anti-mouse IgG, HRP-linked (Cell Signaling, #7076) and anti-Rabbit IgG, HRP-linked (Cell Signaling, #7074) has been cited in at least 3282 and 5961 references, respectively, such as an application in western blots: PMID: 33122197.
Anti-FLAG antibody (Sigma, F1804, M2) has been cited in at least 5374 references such as an application for ChIP: PMID: 24752179.

Eukaryotic cell lines

Policy information about cell lines

Cell line source(s)	HEK293 and N2a cells were obtained from ATCC (Manassas, VA).
Authentication	The cell lines were not authenticated.
Mycoplasma contamination	All the cell lines tested negative for mycoplasma contamination.
Commonly misidentified lines (See ICLAC register)	No such cell line was used

Animals and other organisms

Policy information about studies involving animals; ARRIVE guidelines recommended for reporting animal research

Laboratory animals	Acta1-Cre+ (Stock#006139), Pax7creER/+ (Stock#017763), Col1a1-tetO-4Fhomo (Stock#011004), systemic 4F mice (Stock#011001), ROSA26-LSL-rtTAhomo (Stock#005670), R26-M2rtTA (Stock#006965) and Ai14homo (Stock#007908) mice were from Jackson Laboratory. Col1a1-tetO-4Fhomo mice were mated with ROSA26-LSL-rtTAhomo mice to generate Col1a1-tetO-4FhetROSA26-LSL-rtTAhet (het/het). Het/het mice were mated to generate Col1a1-tetO-4FhomoROSA26-LSL-rtTAhomo (4Fhomo) mice. Acta1-Cre+ and Pax7creER/+ mice were mated with 4Fhomo mice, respectively, to generate Acta1-Cre/4Fhet (representing both Acta1-Cre+/4Fhet and Acta1-Cre-/4Fhet) and Pax7creER/4Fhet (Pax7creER/+/4Fhet and Pax7+/+/4Fhet) mice. Acta1-Cre+/4Fhet and Ai14homo mice were mated to generate Acta1-Cre+/4Fhet/Ai14het mice. We used 6-weeks to 4-month-old as young mice and 12 to 15-month-old as aging mice, both male and female mice for this study.
Wild animals	This study did not involve wild animals.
Field-collected samples	This study did not involve samples collected from field.
Ethics oversight	All animal procedures were performed according to protocols approved by the IACUC and Animal Resources Department of the Salk Institute for Biological Studies.

Note that full information on the approval of the study protocol must also be provided in the manuscript.
